# Genes From a Translational Analysis Support a Multifactorial Nature of White Matter Hyperintensities

**DOI:** 10.1161/STROKEAHA.114.007649

**Published:** 2015-01-26

**Authors:** Lorna M. Lopez, W. David Hill, Sarah E. Harris, Maria Valdes Hernandez, Susana Munoz Maniega, Mark E. Bastin, Emma Bailey, Colin Smith, Martin McBride, John McClure, Delyth Graham, Anna Dominiczak, Qiong Yang, Myriam Fornage, M. Arfan Ikram, Stephanie Debette, Lenore Launer, Joshua C. Bis, Reinhold Schmidt, Sudha Seshadri, David J. Porteous, John Starr, Ian J. Deary, Joanna M. Wardlaw

**Affiliations:** From the Centre for Cognitive Ageing and Cognitive Epidemiology (L.M.L., M.V.H., S.M.M., M.E.B., J.S., I.J.D., J.M.W.), Division of Neuroimaging Sciences, Brain Research Imaging Centre, (M.V.H., S.M.M., M.E.B., J.M.W.) and Academic Neuropathology (C.S.), Centre for Clinical Brain Sciences, University of Edinburgh, Edinburgh, United Kingdom; Centre for Cognitive Ageing and Cognitive Epidemiology, Medical Genetics Section, University of Edinburgh Centre for Genomics and Experimental Medicine and MRC Institute of Genetics and Molecular Medicine, University of Edinburgh, Edinburgh, United Kingdom (W.D.H., S.E.H., D.J.P.); Department of Bioengineering, Imperial College London, London, United Kingdom (E.B.); BHF Glasgow Cardiovascular Research Centre, Institute of Cardiovascular and Medical Sciences, University of Glasgow, Glasgow, United Kingdom (M.M., J.M., D.G., A.D.); Department of Biostatistics, Boston University School of Public Health, MA (Q.Y.); The Framingham Heart Study, Boston, MA (Q.Y., S.S.); The Human Genetics Center and Institute of Molecular Medicine, The University of Texas Health Science Center, Houston (M.F.); Departments of Epidemiology, Radiology and Neurology, Erasmus Medical Center, Rotterdam, The Netherlands (M.A.I.); Netherlands Consortium for Healthy Aging, Leiden, The Netherlands (M.A.I.); 12 INSERM U740 (Paris 7 University) and U708 (Bordeaux University), Bordeaux, France (S.D.); Department of Neurology, Lariboisière Hospital, 7 University, DHU Neurovasc Paris Sorbonne, Paris, France (S.D.); University of Versailles Saint-Quentin-en-Yvelines, Versailles, France (S.D.); Department of Neurology, Boston University School of Medicine, MA (S.D., S.S.); Laboratory of Epidemiology and Population Sciences, Intramural Research Program, National Institute on Aging, National Institutes of Health, Bethesda, MD (L.L.); Cardiovascular Health Research Unit, Department of Medicine, University of Washington, Seattle (J.C.B.); and Clinical Division of Neurogeriatrics, Department of Neurology, Medical University of Graz, Graz, Austria (R.S.).

**Keywords:** genetics, humans, leukoencephalopathies, magnetic resonance imaging

## Abstract

**Background and Purpose—:**

White matter hyperintensities (WMH) of presumed vascular origin increase the risk of stroke and dementia. Despite strong WMH heritability, few gene associations have been identified. Relevant experimental models may be informative.

**Methods—:**

We tested the associations between genes that were differentially expressed in brains of young spontaneously hypertensive stroke–prone rats and human WMH (using volume and visual score) in 621 subjects from the Lothian Birth Cohort 1936 (LBC1936). We then attempted replication in 9361 subjects from the Cohorts for Heart and Aging Research in Genomic Epidemiology (CHARGE). We also tested the subjects from LBC1936 for previous genome-wide WMH associations found in subjects from CHARGE.

**Results—:**

Of 126 spontaneously hypertensive stroke–prone rat genes, 10 were nominally associated with WMH volume or score in subjects from LBC1936, of which 5 (*AFP*, *ALB*, *GNAI1*, *RBM8a*, and *MRPL18*) were associated with both WMH volume and score (*P*<0.05); 2 of the 10 (*XPNPEP1*, *P*=6.7×10^−5^; *FARP1*, *P*=0.024) plus another spontaneously hypertensive stroke–prone rat gene (*USMG5*, *P*=0.00014), on chromosomes 10, 13, and 10 respectively, were associated with WMH in subjects from CHARGE. Gene set enrichment showed significant associations for downregulated spontaneously hypertensive stroke–prone rat genes with WMH in humans. In subjects from LBC1936, we replicated CHARGE’s genome-wide WMH associations on chromosomes 17 (*TRIM65* and *TRIM47*) and, for the first time, 1 (*PMF1*).

**Conclusions—:**

Despite not passing multiple testing thresholds individually, these genes collectively are relevant to known WMH associations, proposed WMH mechanisms, or dementia: associations with Alzheimer's disease, late-life depression, ATP production, osmotic regulation, neurodevelopmental abnormalities, and cognitive impairment. If replicated further, they suggest a multifactorial nature for WMH and argue for more consideration of vascular contributions to dementia.

## Abstract 2

Supplemental Digital Content is available in the text.

White matter hyperintensities (WMH) of presumed vascular origin, a major component of cerebral small vessel disease (SVD), double the risk of stroke and dementia.^1^ Despite considerable societal effect, the causes of WMH and SVD are poorly understood.^2^ Conventional vascular risk factors explain little of the WMH variance.^3^ Family studies,^4^ several rare monogenic SVD disorders,^5^ and epidemiology^6^ suggest that genetic predisposition is important.

Identification of genetic factors for SVD has been challenging. Several replicable single-nucleotide polymorphisms (SNPs) associated with WMH have been identified in 1 locus on chromosome 17q25,^7,8^ although the exact gene(s) and biological pathways to WMH are unclear. Few other replicable genes have been found in genome-wide association studies (GWAS),^9,10^ and little is known of their functional significance.

Experimental SVD models might provide insight into human SVD. The spontaneously hypertensive stroke–prone rat (SHRSP) is a relevant model of spontaneous SVD.^11^ It was selectively crossbred (1974) from Wistar-Kyoto (WKY) rats via the spontaneously hypertensive rat (SHR, 1963).^12^ Hypertension, established in SHRSP rats by 10 weeks of age, is considered to be the main cause of their brain disease. However, differences in protein and gene expression in SHRSP rats versus WKY rats at 5 weeks of age (before measurable blood pressure rises) suggest underlying susceptibilities to SVD.^13^ Compared with WKY controls, 5-week-old SHRSP rats have reduced claudin 5 (tight junction) and myelin basic protein and increased microglia (IBA1) and glial activation (GFAP)^13^; at 16 and 21 weeks, increase in smooth muscle actin was seen, thought to reflect arteriolar smooth muscle hyperplasia secondary to hypertension. SHRSP gene expression differences at 5 weeks of age were more numerous than at 16 or 21 weeks of age and included downregulation of *Mmp14*, *Mbp*, *GFAP*, *AVP*, *Alb*, and *Igf2*, upregulation of *Gucy1A3*, *Rps9*, *Fos*, and *JunB*, early-growth response, cell-signaling genes, and overexpression of genes involved in neurological diseases (stroke, depression, and blood–brain barrier leakage),^14^ rather than just hypertension. Recent gene sequencing of SHRSP rats (and 26 other rat models of common human diseases)^15^ revealed that genes that were either shared between or uniquely mutated in these rat models were significantly over-represented in human GWAS hits for hypertension or metabolism-related phenotypes, suggesting coevolution of these genes and their role in common diseases in models and humans.^15^

In a hypothesis-driven collaborative approach, we tested for associations between genes that were differentially expressed in the brains of 5-week-old SHRSP rats^14^ and WMH in humans. We used data from 5-week-old rats because gene expression differences were more frequent at that age than at 16 or 21 weeks, and we wanted to minimize the confounding of tissue changes by secondary effects of hypertension and to optimize the chances of detecting genes related to WMH susceptibility. We focused on WMH as the most frequent feature of SVD with the most data available in replication cohorts. We first tested the subjects from Lothian Birth Cohort 1936 (LBC1936)^16,17^ and then attempted replication in subjects from the Cohorts for Heart and Aging Research in Genomic Epidemiology (CHARGE) consortium.^7^ To provide confidence in the relevance of subjects from LBC1936, we also sought CHARGE’s^7^ previously reported WMH-gene associations in the subjects from LBC1936.

## Methods

### Subjects

The subjects from LBC1936 are community-dwelling individuals living in South East Scotland who underwent detailed cognitive, biomedical, genetic assessments, and detailed brain MRI at ≈73 years of age (n=866).^16,17^ The MRI acquisition, methods for assessing WMH burden^17^ qualitatively^18^ and quantitatively,^19^ and proportions with WMH by either method^20^ have been reported. This study was approved by the Lothian (REC 07/MRE00/58) and Scottish Multicentre (MREC/01/0/56) Research Ethics Committees; all subjects gave written informed consent.

The subjects from LBC1936 had genome-wide SNP data on 542 050 SNPs,^21^ imputed to 2.5 million SNPs with HapMap2.^22^ There were 621 participants (392 men) from LBC1936 with both MRI and genetic data (mean age, 72.67 years; SD=0.73 years; Table I and Methods in the online-only Data Supplement). We excluded 48 subjects from LBC1936 with a history of stroke or dementia.

### Gene Analysis

In the 5-week-old SHRSP rats, 162 genes were differentially expressed compared with 5-week-old WKY rats in frontal and midcoronal brain sections (Table II in the online-only Data Supplement).^14^ We used the following databases to match the SHRSP Illumina IDs to human genes (Materials and Table II in the online-only Data Supplement): Ensembl—http://www.ensembl.org, GeneCards—http://www.genecards.org, Illumina ID search—http://www.genscript.com, NCBI—http://www.ncbi.nlm.nih.gov, and Rat Genome Database—http://www.rgd.mcw.edu. Of the 162 SHRSP genes, 132 had an equivalent human gene, 8 transcripts were mapped to the same gene, 20 were uncharacterized in humans, and 2 had no human homologue. Of the 132 genes, 126 were available for association testing using the Versatile Gene-based Association Study (VEGAS) test.^23^ We first performed a genome-wide association analysis on subjects from LBC1936 using PLINK software^24^ to test the genetic association between 542 050 genotyped SNPs and 2 WMH measurements using a linear regression analysis: (1) log transformed WMH volume (mL), with age, sex, intracranial volume, and first 4 multiple dimension scaling components for population stratification as covariates; and (2) summed Fazekas score of periventricular and deep WMH, with age, sex, and the first 4 multiple dimension scaling population stratification components as covariates. We used both WMH volume and Fazekas score^20^ to increase the reliability of the results. We did not stratify by vascular risk factors because hypertension (although it was the strongest vascular risk factor) explained <2% of WMH variance in subjects from LBC1936.^3^ The VEGAS software summarized evidence for association with WMH in subjects from LBC1936 per gene by considering the *P* values of all 543 050 SNPs that were located within 17 681 unique autosomal genes (including SNPs±50 kb outside of genes to include regulatory regions). For a more direct comparison with CHARGE (which used imputed data), we also performed a gene-based test on LBC1936’s 2 447 226 HapMap2 derived *P* values (after removing SNPs with a minor allele frequency of <0.01 and imputation quality of <0.3) with VEGAS software as above.

### Replication in Subjects From CHARGE

We then tested whether any of the 126 SHRSP genes were also associated with WMH in subjects from CHARGE by using data from CHARGE’s published genome-wide meta-analysis of WMH in 9361 stroke-free individuals from 7 community-based cohorts.^7^ We performed a gene-based test using VEGAS software, which summarized the evidence for association with WMH burden on a per gene basis, as above, by considering the associated *P* values of all HapMap2 SNPs located within 17 787 autosomal genes (including SNPs±50 kb outside of genes to include regulatory regions).

### Gene Set Enrichment

We performed a gene set enrichment analysis^25^ to investigate the enrichment of the 126 SHRSP genes in the LBC1936 and CHARGE data associated with WMH, accounting for whether these were upregulated or downregulated (online-only Data Supplement),^26^ corrected for multiple testing using a false discovery rate (FDR) method.^27^

### Replication of Previous CHARGE Findings in Subjects From LBC1936

To demonstrate our ability to detect WMH-gene associations in subjects from LBC1936, we attempted replication of CHARGE’s genome-wide associations with WMH^7,8^ in the subjects from the LBC1936 Cohort in a genome-wide association analysis using the 2 534 887 SNPs imputed to HapMap2, with WMH (volume and Fazekas score) in Mach2QTL software.^28^

We applied Bonferroni correction for multiple testing (*P*=0.05/126 genes=0.0004). We did not include the 2 WMH phenotypes in the Bonferroni correction as they are highly correlated (*r*^2^=0.77). Because of the overconservative nature of Bonferroni correction for multiple testing,^29^ a nominal significance threshold of *P* value of <0.05 was required for replication efforts.

## Results

### SHRSP Genes in Subjects From LBC1936

Of the 126 candidate SHRSP-derived genes, 10 were nominally associated with WMH in subjects from LBC1936 (*P*<0.05; Table 1). Using imputed or genotyped data, 5 genes were associated with WMH volume (*AFP*, *ALB*, *GNAI1* [*RBM8A* and *INPP5D*, both borderline]); 3 of these (*AFP*, *ALB*, and *GNAI1*) and 2 others (*MRPL18* and *SIPA1L2*) were associated with WMH Fazekas scores. Three other genes were associated with WMH volume using genotyped data only (*XNXPEP1*, *NR4A3*, and *FARP1*). None of these genes individually passed Bonferroni correction in subjects from LBC1936 (all were *P*>0.0004), in part, reflecting the LBC1936 sample size.

**Table 1.**
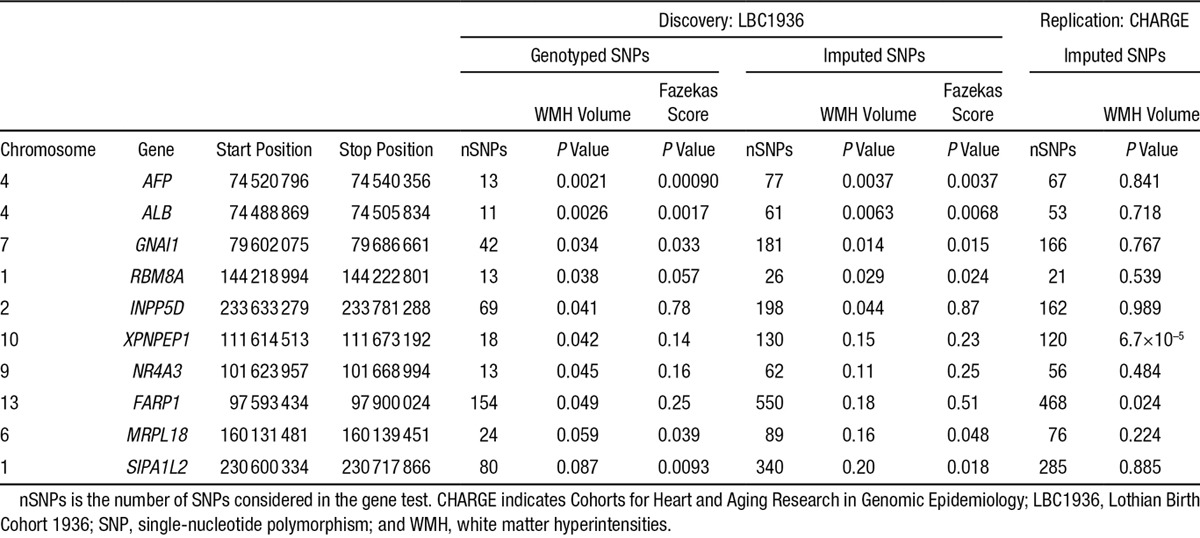
Genes Associated With Cerebral Small Vessel Disease in Rats That Are Associated With WMH in Older Humans: 126 Differentially Expressed Genes Between Spontaneously Hypertensive Stroke Prone and Wild-Type Rats Were Tested for Association With WMH in Subjects From LBC1936 and 10 Genes Were Significantly Associated (P<0.05) With Either WMH Volume or Fazekas Score

### SHRSP Genes in Subjects From CHARGE

Two of these 10 genes were also associated with WMH in subjects from CHARGE (*XPNPEP1*, *P*=6.7×10^−5^; and *FARP1*, *P*=0.024; Table 1). Full details of all 126 SHRSP to LBC1936 to CHARGE gene associations are given in Table III in the online-only Data Supplement. Several other of the 126 SHRSP genes (outside the 10/126 described above) showed significance at *P*<0.05 in subjects from CHARGE (eg, *USMG5*, *MED17*, *ZNF461*, *C20orf7*, *EGR1*, *ARC*, *NUDT14*, and *MMP14*) of which 1 (*USMG5*, *P*<0.000142) passed Bonferroni correction (*P*<0.0004).

### Gene Set Enrichment

Using gene set enrichment analysis, all 126 SHRSP candidate genes were not enriched in subjects from LBC1936 for association with WMH in the 17 681 genes tested here (WMH volume, *P*=0.34; Fazekas score, *P*=0.81), but this would not preclude the possibility that in either upregulated or downregulated gene sets, there was an abundance of genes showing an enriched association. We tested the upregulated (n=76) and downregulated (n=50) SHRSP genes separately and found significant enrichment for Fazekas scores in SHRSP downregulated genes (*P*=0.035; FDR, 0.046) but not SHRSP upregulated genes (*P*=0.921; FDR, 0.899). WMH volume showed significant enrichment in downregulated (*P*=0.018; FDR, 0.025) but not upregulated (*P*=0.802; FDR, 0.780) genes. In the CHARGE consortium, there was no significant enrichment for either the total set of 126 genes (*P*=0.0514), the upregulated (*P*=0.109; FDR, 0.266) or the downregulated genes (*P*=0.173; FDR, 0.149).

### Replication of CHARGE’s Previous Genome-Wide Association in Subjects From LBC1936

We sought CHARGE’s previous genome-wide association results for WMH^7^ in subjects from LBC1936. Of CHARGE’s 15 SNPs (*P*<1×10^−5^) associated with WMH (Table 2),^7^ 3 SNPs replicated in subjects from LBC1936 with both WMH volume and Fazekas score at *P*<0.05 (rs3744028, rs1055129, and rs1052053); rs1052053, a miss-sense variant on chromosome 1 in the polyamine-modulated factor 1 gene (*PMF1*), has not replicated previously.

**Table 2.**
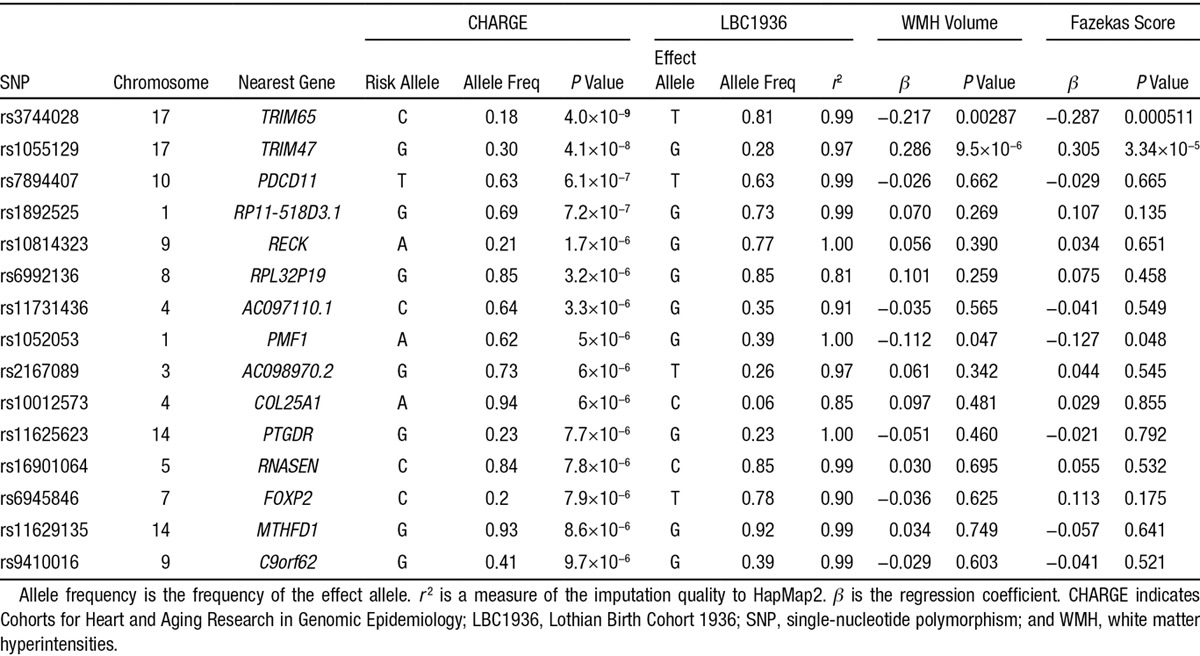
Association of SNPs Previously Associated With WMH in CHARGE in Subjects From LBC1936 and the Corresponding SNP Association Results Are Given for LBC1936 WMH Volume and Fazekas Score

## Discussion

We used a clinically relevant translational approach^15^ to identify potential new gene associations for WMH, a common cause of cognitive impairment, stroke, and dementia. We found parallels between differentially expressed genes in a young spontaneous SVD model and WMH-gene associations in older humans. Two novel genes on chromosome 10 derived from SHRSP rats were associated with WMH, *XPNPEP1* in both LBC1936 and CHARGE and *USMG5* in CHARGE only. Several other genes were nominally associated with WMH in LBC1936 or CHARGE although none passed multiple testing. We replicated 3 of CHARGE’s WMH-gene associations in subjects from LBC1936: 2 (rs3744028 and rs1055129) on chromosome 17q25 and 1 previously unreplicated SNP (rs1052053) on chromosome 1, a miss-sense variant in the polyamine-modulated factor 1 gene, *PMF1*, that has a role in the cell cycle. Jointly, these approaches yielded 6 genes (3 from the SHRSP rats and 3 replicates of a GWAS finding) and 5 further rat-derived genes based on the LBC1936 sample alone, which despite not passing multiple testing thresholds individually, as a group they are notable for their involvement in biological pathways relevant to WMH pathogenesis.^2^

Of the 2 SHRSP genes found in LBC1936 and CHARGE, *XPNPEP1* is X-prolyl aminopeptidase (aminopeptidase P) 1, soluble, associated with biliary atresia, and located in a region on chromosome 10 that is associated with Alzheimer's disease.^30^ *FARP1* is Pleckstrin domain protein 1, associated with brain volume differences,^31^ and important in synapse development.^32^ The SHRSP-CHARGE–associated gene *USMG5* is upregulated during skeletal muscle growth 5 homolog (also known as diabetes mellitus–associated protein in insulin sensitive tissues, or *DAPIT*), sits on chromosome 10, and maintains ATP synthase populations in mitochondria.^33^ All 5 SHRSP genes associated with both WMH volume and Fazekas score in subjects from LBC1936 (*AFP*, *ALB*, *GNAI1*, *RBM8A*, and *MRPL18*) are associated with white matter–relevant diseases in humans. Despite not surviving correction for multiple testing, there was a notable consistency in their association with 2 separate WMH measures. *AFP* encodes α-fetoprotein, a major plasma protein produced in the yolk sac and liver during fetal life. Abnormally, high amounts of α-fetoprotein are found in ataxia telangiectasia,^34^ also associated with abnormal white matter.^35^ *ALB* encodes albumin, a soluble monomeric protein important for maintaining plasma oncotic pressure found in cerebral WMH,^36^ and cerebrospinal fluid as blood–brain barrier function deteriorates with ageing and dementia.^2,37^ *GNAI1* encodes guanine nucleotide–binding protein (G protein), alpha-inhibiting activity polypeptide 1, implicated with Alzheimer's disease.^38^ *RBM8A* is an RNA binding protein that has differential expression in Alzheimer's disease,^39^ associations with a range of intellectual disabilities in humans and anxiety-related behavior in mice,^40^ with schizophrenia, several neurodevelopmental intellectual disabilities, anxiety behavior and may target neuronal genes to regulate behaviors. WMH in old age are known associates of late-onset depression,^41^ and they are also associated with lower age 11 IQ.^42^ *MRPL18* is the mitochondrial ribosomal protein L18, previously associated with multiple sclerosis.^43^ These 7 SHRSP-derived genes are related to pathologies (ataxia telangiectasia, blood–brain barrier impairment, Alzheimer's disease, multiple sclerosis, depression, developmental intellectual disabilities, and brain size) that display white matter abnormalities or affect intellectual function. Impaired ATP production because of defects in *USMG5*, the gene that replicated from SHRSP to CHARGE, could increase susceptibility to WMH via ischemia.

The genes that were downregulated in the SHRSP were significantly enriched in subjects from LBC1936 for WMH. This may be because, in a complex disease such as SVD/WMH, several individually modest genetic defects in different components of key pathways, when present in combination, increase disease risk. This interpretation is consistent with differential protein expression seen in SHRSP^13^ and the absence, so far, of individual major human gene defects explaining either sporadic WMH or lacunar stroke.^9^

The lack of consistent replication from SHRSP to LBC1936 to CHARGE requires caution. The power and required significance threshold of the LBC1936 was modest for GWAS, hence our hypothesis-driven approach. Genes associated with WMH in subjects from LBC1936 but not CHARGE could be false positives; other factors include greater heterogeneity of WMH assessment and greater age range in subjects from CHARGE. The narrow age range of subjects from LBC1936 minimizes the effect of age, possibly helping to expose relevant genes. CHARGE-contributing studies used several methods of quantifying WMH, different MR scanner field strengths, and generations of technology and sequences. However, WMH volume and visual scores are highly correlated,^20^ and our replication of 3 findings from CHARGE in subjects from LBC1936 suggests that our approach has some validity. The CHARGE cohorts may have used different imputation platforms or more SNPS may have failed quality assurance in subjects from LBC1936, contributing to differences between the imputation results. There are several limitations to gene-based analysis, including the omission of nonautosomal genes, the effect of noncausal SNPs to dilute association (in particular, in the presence of a strong genetic association with a single locus within or in the regulatory region of a given gene, thus missing important associations), the lack of knowledge on (and overlap of) gene boundaries, the possibility that an SNP variant may influence a gene distal to its site, thus not corresponding to a gene that it is located next to it, and the potential of the genetic data not to tag causative genetic variants. Power may have been limited (despite CHARGE’s large sample size) to detect associations with some genes. We did not stratify the human cohorts by risk factors as these explained <2% of WMH variance in subjects from LBC1936,^3^ and risk-stratified genetic data were unavailable for CHARGE. We did not test gene associations with other SVD features in addition to WMH because a total SVD burden score was not available for CHARGE. Although it is a relevant model of spontaneous SVD^11,12^ and of human hypertension and metabolic disorders,^15^ like any model, the SHRSP has translational limitations, arguing for additional studies at different ages and brain regions, with or without environmental stressors.

This work has the following strengths: accurate LBC1936 WMH phenotyping^17^ and genetic information in this relatively large narrow age-range older population.^16^ The Glasgow SHRSP colony is long established, with carefully controlled environments. The mRNA data were obtained from the same rats that provided protein expression data.^13^ Replication in other SHRSP colonies and examination of related strains (eg, SHR’s) may be informative. The genomes of SHRSP and 26 other complex disease phenotype models were recently sequenced,^15^ showing associations between genes in rat models of hypertension and human GWAS hits for hypertension phenotypes.^15^ This provides support for our reverse-translational discovery approach, suggesting that genes in disease models have coevolved and may contribute to disease-related phenotypes in humans.

Our findings require validation. The selection of candidate genes for investigation could be widened by examining more genes from the 5-week-old SHRSP rats (Table II in the online-only Data Supplement), other models,^15^ and in larger samples of well-phenotyped humans, such as from METASTROKE and the Wellcome Trust Case-Control Consortium. This translational analysis of experimental models and human disease suggests some aspects of the genetic architecture underlying SVD, stroke, and dementia and argues for greater awareness of vascular contributions to neurodegeneration.

Figure I and Tables IV and V in the online-only Data Supplement provide the top SNP (*P*<1×10^−5^) and gene (*P*<0.001) associations with WMH variables in subjects from LBC1936 for further reference.

## Acknowledgments

We thank the Lothian Birth Cohort 1936 participants and research team members, Wellcome Trust Clinical Research Facility (http://www.wtcrf.ed.ac.uk, subject testing and genotyping), and Brain Research Imaging Centre (http://www.bric.ed.ac.uk, brain imaging and analyses). Cohorts for Heart and Aging Research in Genomic Epidemiology thanks the staff and participants of the Aging Gene-Environment Susceptibility-Reykjavik Study, Atherosclerosis Risk in Community Study (ARIC), Austrian Stroke Prevention Study (ASPS), Cardiovascular Health Study, and Framingham Heart and Rotterdam Studies for their important contributions. ASPS thanks Birgit Reinhart for her long-term administrative commitment and Ing Johann Semmler for the technical assistance at creating the DNA bank. Drs Wardlaw, Bailey, McBride, Graham, Dominiczak, Deary, Starr, Seshadri, Fornage, Ikram, Debette, Launer, Bis, and Schmidt contributed to data collection. Dr Lopez, Harris, Hill, Yang, Bailey, McClure, McBride, Smith, Hernandez, Maniega, Bastin, and Wardlaw contributed to data analysis. Drs Wardlaw, Deary, and Seshadri contributed to study design, co-ordination, and funding. Lopez, Wardlaw, Seshadri, and Deary contributed to article preparation. Lopez, Harris, Hill, Porteous, Smith, Deary, Starr, Seshadri, Yang, Fornage, Ikram, Debette Launer, Bis, Schmidt, Bailey, McBride, Graham, McClure, Dominiczak, Hernandez, Maniega, Bastin, and Wardlaw contributed to article review. Wardlaw was the guarantor and provided the overall concept.

## Sources of Funding

Lothian Birth Cohort 1936 was funded by Age UK’s Disconnected Mind programme (http://www.disconnectedmind.ed.ac.uk) and by Research Into Ageing (references 251 and 285). Whole-genome association was funded by Biotechnology and Biological Sciences Research Council (reference BB/F019394/1), brain image analysis was funded by Medical Research Council (G1001401 and 8200), and imaging was funded by Brain Research Imaging Centre (http://www.bric.ed.ac.uk), The University of Edinburgh Centre for Cognitive Ageing and Cognitive Epidemiology (http://www.ccace.ed.ac.uk, G0700704/84698). The SHRSP analysis was funded by Medical Research Council (Dr Bailey), British Neuropathological Society, Newby Fund, Scottish Funding Council Scottish Imaging Network A Platform for Scientific Excellence Collaboration (http://www.sinapse.ac.uk), and European Union Community’s FP7/2007–2013, grant agreement HEALTH-F4-2010-241504 EURATRANS. The Aging Gene-Environment Susceptibility-Reykjavik Study was funded by National Institutes of Ageing (NIA), contract N01-AG-12100, National Eye Institute, National Institute on Deafness and Other Communication Disorders, National Heart, Lung, and Blood Institute (NHLBI), the NIA Intramural Research Program, Hjartavernd (the Icelandic Heart Association), and the Althingi (the Icelandic Parliament). The Atherosclerosis Risk in Communities Study is a collaborative study supported by NHLBI contracts (HHSN268201100005C, HHSN268201100006C, HHSN268201100007C, HHSN268201100008C, HHSN268201100009C, HHSN268201100010C, and HHSN268201100011C, HHSN268201100012C), R01HL087641, R01HL59367 and R01HL086694; National Human Genome Research Institute contract U01HG004402; and National Institutes of Health (NIH) contract HHSN268200625226C; NIH grant number UL1RR025005, and NIH Roadmap for Medical Research and grant number HL093029. The Austrian Stroke Prevention Study was funded by the Austrian Science Fond grant numbers P20545-P05 and P13180. The Medical University of Graz supports the databank of the ASPS. The Cardiovascular Health Study was supported by NHLBI contracts N01-HC-85239, N01-HC-85079 through N01-HC-85086, N01-HC-35129, N01 HC-15103, N01 HC-55222, N01-HC-75150, N01-HC-45133, HHSN268200960009C, and HHSN268201200036C and NHLBI grants HL080295, HL087652, HL105756 with additional contribution from National Institute of Neurological Disorders and Stroke, AG-023629, AG-15928, AG-20098, and AG-027058 from the NIA (http://www.chs-nhlbi.org/pi.htm). DNA handling and genotyping at Cedars-Sinai Medical Center was supported in part by Cedars-Sinai Board of Governors’ Chair in Medical Genetics (JIR), the National Center for Research Resources, grant UL1RR033176, and is now at the National Center for Advancing Translational Sciences, Clinical and Translational Science Institute grant UL1TR000124; in addition to the National Institute of Diabetes and Digestive and Kidney Disease grant DK063491 to the Southern California Diabetes Endocrinology Research Center. The Framingham Heart Study was supported by the NHLBI (Contract no. N01-HC-25195) and its contract with Affymetrix, Inc for genotyping services (Contract no. N02-HL-6-4278); some of this research used the Linux Cluster for Genetic Analysis (LinGA-II) funded by the Robert Dawson Evans Endowment of the Department of Medicine at Boston University School of Medicine and Boston Medical Center; and grants from the National Institute of Neurological Disorders and Stroke (R01 NS17950), and the National Institute of Aging (R01s AG08122, AG16495, and AG033193), and the NHLBI (U01 HL096917 and R01 HL093029). The Rotterdam Study Genome-Wide Association database was funded by the Netherlands Organization of Scientific Research (NOW; no. 175.010.2005.011); the study was further supported by the Netherlands Genomics Initiative/NWO project no. 050-060-810; the Erasmus Medical Center and Erasmus University, Rotterdam; NWO, the Netherlands Organization for the Health Research and Development, the Research Institute for Diseases in the Elderly, the Ministry of Education, Culture and Science, the Ministry for Health, Welfare and Sports, the European Commission (Dr Graham, XII), and the Municipality of Rotterdam and Dr Ikram was supported by the Nederlandse Hartstichting grant 2009B102 and ZonMW Veni-grant 916.13.054.

## Disclosures

None.
